# Short-Term High-Intensity Interval Training on Body Composition and Blood Glucose in Overweight and Obese Young Women

**DOI:** 10.1155/2016/4073618

**Published:** 2016-09-28

**Authors:** Zhaowei Kong, Shengyan Sun, Min Liu, Qingde Shi

**Affiliations:** ^1^Faculty of Education, University of Macau, Macau; ^2^Institute of Physical Education, Huzhou University, Huzhou, Zhejiang, China; ^3^School of Physical Education and Sports, Macau Polytechnic Institute, Macau

## Abstract

This study was to determine the effects of five-week high-intensity interval training (HIIT) on cardiorespiratory fitness, body composition, blood glucose, and relevant systemic hormones when compared to moderate-intensity continuous training (MICT) in overweight and obese young women.* Methods*. Eighteen subjects completed 20 sessions of HIIT or MICT for five weeks. HIIT involved 60 × 8 s cycling at ~90% of peak oxygen consumption ($\dot{V}O_{2peak}$) interspersed with 12 s recovery, whereas MICT involved 40-minute continuous cycling at 65% of $\dot{V}O_{2peak}$. $\dot{V}O_{2peak}$, body composition, blood glucose, and fasting serum hormones, including leptin, growth hormone, testosterone, cortisol, and fibroblast growth factor 21, were measured before and after training.* Results*. Both exercise groups achieved significant improvements in $\dot{V}O_{2peak}$ (+7.9% in HIIT versus +11.7% in MICT) and peak power output (+13.8% in HIIT versus +21.9% in MICT) despite no training effects on body composition or the relevant systemic hormones. Blood glucose tended to be decreased after the intervention (*p* = 0.062). The rating of perceived exertion in MICT was higher than that in HIIT (*p* = 0.042).* Conclusion*. Compared with MICT, short-term HIIT is more time-efficient and is perceived as being easier for improving cardiorespiratory fitness and fasting blood glucose for overweight and obese young women.

## 1. Introduction

Obesity has become a major global health challenge. Although epidemiological data show that regular physical activity helps prevent obesity, cardiovascular disease, diabetes, and hypertension [1], the majority of adults fail to maintain physical activity at levels that can promote health. Given that “lack of time” is the most commonly cited barrier to regular exercise participation [2], a more time-efficient mode of exercise training has been developed. Both high-intensity interval training (HIIT; involves “near maximal” effort at intensities between 80 and 100% of maximal heart rate) [3, 4] and low-volume sprint interval training (SIT; involves “all-out” or “supramaximal” effort at the intensity of 100% of maximal oxygen uptake) [3, 4] are time-efficient training strategies that can rapidly improve cardiorespiratory fitness [5, 6], muscle metabolic adaptations [7, 8], and insulin sensitivity [5] to resemble the changes elicited by moderate-intensity continuous training (MICT).

Excessive accumulation of body fat, especially intra-abdominal visceral fat, is identified as a potent independent predictor for hyperlipidemia, insulin resistance, and metabolic syndrome, whereas a slight change in the visceral adipose area/volume may significantly alter the risk profile [9]. A growing body of evidence has demonstrated that HIIT/SIT could reduce body mass [5, 10], total or regional fat mass [6, 10–12], and waist circumference [10, 11, 13] and increase fat-free mass [5, 6, 10–12]. However, in terms of short-term intervention (e.g., 18 sessions for six weeks), it seems that SIT running is effective at improving body composition in active individuals [6, 11], whereas HIIT interventions showed inconsistent findings in terms of body composition, including improvement [12] or ineffectiveness [14, 15]. From the view of the influential factors of body composition, confounding factors such as extra physical activity in addition to the training program [5, 10, 11] and/or the dietary intake of the participants [6, 8, 13, 16] have been neglected in some studies. Given the conflicting results in previous studies [5, 17], whether the HIIT intervention induces significant improvements in fat mass, lean mass, and regional fat deposits in comparison with an exercise control remains to be elucidated.

Although some studies have reported that HIIT protocols are enjoyable and adherent for both active male subjects [18] and inactive normal-weight subjects [19], some researchers have contended that, for the largely inactive and/or obese population, the strenuous nature of SIT may produce negative emotions toward exercise adherence and is likely to be a deterrent to participation [18, 20]. Given the fact that the power output of the Wingate-based protocol with hard resistance (typically 7.5% of body mass) rises quickly and then decreases precipitously over the 30 s [7], this kind of SIT training may not be suitable for the inactive female cohort. As a result, relatively less rigorous interval training protocols have been developed. Little et al. [21] designed a low-demanding HIIT model consisting of 10 × 60 s work bouts at 100% maximal heart rate (HR) interspersed with 60 s of recovery. Trapp et al. [5] established a brief HIIT protocol of 8 s sprint cycling interspersed with 12 s of rest for 20 min and found that 15 weeks of HIIT intervention with this protocol could improve cardiovascular fitness, body composition, and insulin resistance in young women when compared to 40 min of MICT with a similar energy expenditure. Given that the young women with a higher BMI had a greater fat loss in Trapp et al.'s study [5], we speculated that overweight/obese women and lean women may have different physiological responses to the same HIIT training protocol.

Hormonal abnormalities play a pathogenetic role in the development of excess body fat and are associated with metabolic diseases [22]. A number of hormones, for example, leptin, growth hormone (GH), testosterone, cortisol, and fibroblast growth factor 21 (FGF-21), regulate lipid metabolism [23] and affect muscle protein synthesis and muscle hypertrophy [24]. It has been reported that acute high-intensity interval exercise may result in optimal responses on circulating testosterone [25], growth hormone [26], and cortisol [26] in well-trained males as well as in type 1 diabetic individuals [27]. However, it is not clear whether the hormonal responses to an acute bout of high-intensity interval exercise can be sustained after a period of training.

Collectively, the purpose of this study was mainly to compare the effects of five weeks of HIIT intervention or MICT on body composition and blood glucose as well as the systemic hormones that may influence body composition and blood glucose in overweight and obese young women. We hypothesized that both training programs would result in similar influences on the improvements of body composition (e.g., reduce fat mass and increase lean mass) and blood glucose, while the improvements are associated with the upregulated GH and testosterone and the downregulated cortisol, leptin, and FGF-21. Furthermore, HIIT would be more time-efficient and perceived as being easier when compared with MICT.

## 2. Method

### 2.1. Participants

The research ethics committee of the University of Macau approved this study. Volunteers were publicly recruited via local media. The inclusion criteria were being between 18 and 30 years of age, having a classification of inactivity (defined as completion of less than 90 min of moderate-intensity exercise per week over the past six months), and being overweight or obese, defined as having a body mass index (BMI, in kg·m^−2^) over 23 and a body fat percentage (%) over 30 [28]. Volunteers who were interested in the study and met the inclusion criteria were required to complete a PAR-Q form and a medical history questionnaire for further eligibility screening. Smokers, alcoholics, diabetics, persons with endocrine disorders, and users of oral contraceptive pills or any prescribed medications known to affect body composition or the endocrine system were excluded. Then, the subjects underwent a full physical examination to obtain clearance for undertaking vigorous exercise from a doctor.

**Figure 1:**
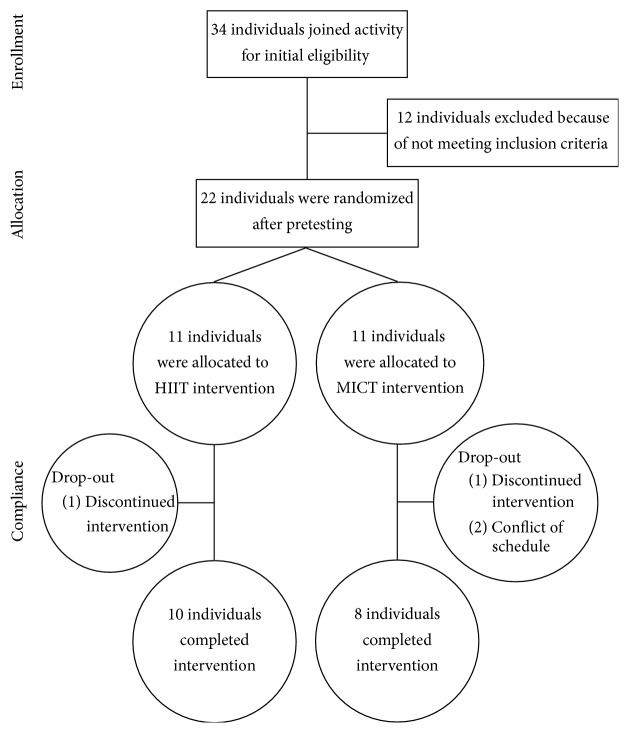
Flow of participants through the intervention of the study.

Under the assumptions of a within-subject correlation of 0.70 between the pre- and postintervention measures and a power of 0.80 with an effect size of 0.48 based on the primary outcome of $\dot{V}O_{2peak}$ resulting from high-intensity interval training [3, 29], the sample size for the HIIT group was estimated to be eight. After the screening phase, 22 eligible subjects were recruited to participate in this study. They all provided written informed consent before being randomly assigned to either the HIIT group (*n* = 11) or the MICT group (*n* = 11). One participant in the HIIT group and three participants in the MICT group quit before completing the training intervention for personal reasons (Figure 1).

### 2.2. Experimental Procedures

Each subject completed a five-week HIIT or MICT exercise intervention (four sessions per week). Before and after the training intervention, subjects underwent a body composition analysis and a $\dot{V}O_{2peak}$ assessment, and their fasting blood samples were obtained. All pretraining and posttraining measures were conducted in the follicular stage based on the self-reported menstrual cycle survey. Because of the different menstrual cycles for the subjects, the starting time for baseline measures and training intervention were different among subjects, with the last subject starting two weeks later than the first one. However, all of the pretraining measures were taken within 48 to 144 h before the training intervention and all of the posttraining measures were taken within 48 to 144 h after the last training session.

### 2.3. Pretraining Testing Protocol

Before baseline measures, each subject visited the laboratory to sign the consent form, become familiar with all testing and training procedures, and provide a three-day diet record. Baseline measures were conducted on three different days, separated by at least 24 h, and all measures were completed at least 48 h before the training intervention. Baseline measures consisted of three tests.

*(1) Baseline Blood Sampling*. Baseline blood samples were taken according to the individuals' menstrual status. Subjects were asked to refrain from strenuous activity and caffeine for 48 h and fasted overnight (12 h) prior to the baseline blood sampling. 8 mL blood samples were collected from the cubital veins using serum separation tubes and were left to clot at room temperature for 60 min. Then, the blood samples were centrifuged at 3000 rpm for 10 min at 4°C, separated for serum, and subsequently frozen at −80°C until later analysis.

*(2) Anthropometrics and Body Composition Assessment*. Subjects were instructed to come to the laboratory in the morning after a fasting state (12 h). Height and weight were determined using standard methods with a stadiometer and an electronic scale (in light clothing and with no footwear) to the nearest 0.1 cm and 0.1 kg, respectively. Body mass index was calculated by dividing weight (kg) by height (m) squared. By the same operator, subjects were scanned in a supine position using a dual-energy X-ray absorptiometry scanner (Norland XR-36 DXA densitometer, Norland Corporation, Fort Atkinson, WS, USA) and were analyzed with a software program (3.7.4/2.1.0; Norland Corporation). The instrument was calibrated daily using the phantoms provided by the manufacturer, and the value of the intra-assay coefficient of variation (CV) was 0.53%. The abdominal region referred to the area consisting of the line between the two iliac crests, the two edges of the hip, and the lateral sides of the femoral necks. The trunk region was defined as being from the lower edge of the mandibular to the upper edge of the line between the iliac crests, excluding the head and upper limbs. Lean mass and fat mass were calculated from the total and regional analysis of the whole body scan.

*(3)* $\dot{V}$ *O*_*2peak*_ *Test*. The subjects performed a graded maximal exercise test on a computer-controlled cycle ergometer (Monark 839E, Sweden) to determine $\dot{V}O_{2peak}$ and peak power output (PPO). After a two-minute warm-up at 30 W, the subjects pedaled at the initial workload of 50 W and maintained a cycling speed of 60 ± 5 rpm, and the workload was increased by 25 W every three min until volitional exhaustion. Respiratory gases were assessed continuously using an automatic gas analyzer (Meta-Max 3B, Cortex Biophysik GmbH, Leipzig, Germany), and the highest oxygen consumption averaged over the final 15 s was identified as the $\dot{V}O_{2peak}$ [30]. PPO was calculated according to the following formula: PPO = *W*_com_ + (*t*/180) × 25, where *W*_com_ is the last completed workload, *t* is the time completed in the final unfinished workload (in seconds), 180 is the increment duration (s) in each workload, and 25 is the workload increment.

### 2.4. Exercise Training

Exercise training commenced at least 48 h after the last baseline measure. Both the HIIT and the MICT exercise training were conducted four days per week for five weeks.

Participants in the HIIT group performed 60 repetitions of high-intensity interval exercise (8 s cycling and 12 s passive recovery) on a cycle ergometer (Monark 874E, Sweden) for 20 min. A prerecorded tape was used to coordinate the HIIT intervention, and all subjects worked as hard as they could during the exercise phase. The initial resistance of the exercise phase was 1.0 kg, and once an individual could complete two consecutive sessions at the given workload, resistance would be gradually increased by increments of 0.5 kg until reaching 0.05 × body weight. HR (Polar F4M BLK, Finland) and ratings of perceived exertion (RPE, Borg scale) were recorded before and immediately after the completion of the 8 s cycling exercise for every five intervals. During the first and last training sessions, the metabolic cart was used to measure energy expenditure. The product of all intervention sessions and the mean value of energy expenditure measured during the two mentioned sessions were regarded as the total energy expenditure of HIIT.

Participants in the MICT group performed a continuous cycling exercise at 65% of pre-$\dot{V}O_{2peak}$ on an ergocycle (Ergometer 900PC, Ergoline, Germany) for 40 min; a cycling speed of 60 ± 5 rpm would be maintained throughout each training session. With the increasing fitness indicated by a decreased HR, the resistance was gradually increased by 0.5 kg. HR and RPE were monitored and continuously recorded every five min. The energy expenditure for every training session was estimated from an individual's $\dot{V}O_{2}$: energy expenditure = 5.05 (kcal·L^−1^) ×  $\dot{V}O_{2}$ (L·min^−1^) × exercise time (min), whereas $\dot{V}O_{2}$ was determined using an equation for leg cycling ergometry: $\dot{V}O_{2}$ = 7.0 + 1.8 × workload (kg·m·min^−1^)/body weight (kg) [31]. Exercise time and workload were recorded during the whole training intervention. The total energy expenditure of the MICT group was calculated as the product of all intervention sessions and the energy expenditure of each session.

### 2.5. Blood Assays

Serum glucose was measured via the glucose oxidase method using a Roche/Hitachi P800 Modular Chemistry Analyzer (Roche Diagnostics GmbH, Mannheim, Germany). Serum concentrations of testosterone, cortisol, and GH were analyzed using commercially available electrochemiluminescence immunoassay kits (Roche Diagnostics GmbH, Mannheim, Germany), whereas leptin and FGF-21 were measured using a commercial enzyme-linked immunosorbent assays (ELISA) kit (Abcam, Cambridge, UK). The CVs were 1.1% for glucose, 4.5% for testosterone, 4.6% for cortisol, 2.9% for GH, 2.4% for leptin, and 2.6% for FGF-21. All analyses were performed using standard procedures (Kingmed Diagnostics Co., Ltd., Guangzhou, China).

### 2.6. Record of Diet and Daily Physical Activity

Subjects in both the HIIT and MICT groups were instructed to maintain their normal eating habits and normal daily physical activities during the study period. Each subject provided a three-day diet inventory one week before and one week after the intervention as well as during the third week of the intervention. Energy intake and diet component analyses were conducted by the Sports Nutrition Research Center (National Institute of Sports Medicine, China) using the nutrition analysis and management system. Daily physical activities were monitored using pedometers (Yamax SW-200 digiwalker, Japan) for three days per week for a total of seven weeks (on the weeks before and after training and every week during exercise training).

### 2.7. Posttraining Testing Protocol

Posttraining assessments were performed in the same way as described in the pretraining testing protocol and were completed within 48 to 144 h following the last training session. Blood samples were taken within 96 to 144 h after the intervention, whereas body composition and $\dot{V}O_{2peak}$ were determined within 48 to 72 h after the last training session. The last subject finished all procedures two weeks after the first one.

### 2.8. Statistical Analysis

Data were analyzed using PASW software (Release 22.0; IBM, NY, USA). The Shapiro-Wilk test was used to assess normality distribution in outcome variables. Independent-sample* t*-tests were performed to determine the differences in training data (HR and RPE) and energy expenditure between the two groups. A two-way mixed analysis of variance (ANOVA) with repeated measures was used to test for main (time) and interaction effects (time × group). Significant interactions or main effects were determined using Tukey's honestly significant difference* post hoc* test. As for effect size measure of the main effect and the interaction effect, partial *η* ^2^ was considered small if *η* ^2^ < 0.06 and large if *η* ^2^ > 0.14 [32]. All results were presented as mean ± standard deviation (SD), and *p* values of < 0.05 were considered significant.

## 3. Results

There were no significant differences on any measured variables between the two groups on pretraining tests.

### 3.1. Training Data

There was no significant difference in training HR between HIIT and MICT (164 ± 8 bpm in the HIIT group versus 160 ± 12 bpm in the MICT group; *p* = 0.435). However, MICT is perceived to be significantly harder compared to HIIT (13 ± 1 in HIIT group versus 15 ± 1 in MICT group; *p* = 0.042).

### 3.2. Energy Expenditure of the Intervention

In the first training session, the values of energy expenditure were 174 ± 28 kcal in HIIT and 301 ± 45 kcal in MICT, and the former spent less than the latter (*p* < 0.001). Similarly, the total energy expenditure of the intervention in the HIIT group (3167 ± 549 kcal) was significantly lower than that in the MICT group (6011 ± 505 kcal; *p* < 0.001).

### 3.3. Diet and Extra Physical Activities

The daily calorie intakes (Table 3) were not different within group and between groups over time (as evaluated before training, during training, and after training; *p* > 0.05). The proportions of macronutrient intake were approximately 50%, 35%, and 15% for carbohydrates, fat, and protein, respectively, in both groups, with no within-group or interaction differences (*p* > 0.05). Physical activities recorded by the pedometers had no within-group or interaction differences before (7673 ± 1145 steps in HIIT versus 8062 ± 1367 steps in MICT), during (9785 ± 1640 steps in HIIT versus 8517 ± 791 steps in MICT), and after intervention (7434 ± 1225 steps in HIIT versus 7023 ± 849 steps in MICT) (*p* > 0.05).

### 3.4. Cardiorespiratory Fitness

After five weeks of exercise training, both HIIT and MICT resulted in a significant improvement in $\dot{V}O_{2peak}$ (*p* = 0.006; *η* ^2^ = 0.38) and PPO (*p* < 0.001; *η* ^2^ = 0.61). HIIT training increased $\dot{V}O_{2peak}$ and PPO by 7.9% and 13.8%, respectively, whereas MICT training increased $\dot{V}O_{2peak}$ and PPO by 11.7% and 21.9%, respectively. There were no group differences in the magnitude of improvement in $\dot{V}O_{2peak}$ and PPO (*p* > 0.05) (Table 1).

### 3.5. Body Composition

After the intervention, despite no significant changes in weight, BMI, total fat mass (TFM), and total body fatness (TBF) for both groups, the MICT group experienced significantly decreased total lean mass (TLM) (−1.7 kg or −3.9%; *p* = 0.011) and leg LM (−0.6 kg or −3.3%, *p* = 0.018). Meanwhile, TLM and leg LM in the HIIT group were unchanged (reduced by −0.2% and −0.1%, resp.; *p* > 0.05). In the regions of the trunk and abdomen, no significant changes in lean mass, fat mass, and fatness were observed within group or between groups (Table 1).

### 3.6. Fasting Glucose and Serum Hormones

Fasting glucose tended to be significantly decreased (*p* = 0.062; *η* ^2^ = 0.213) after the training intervention, but no group difference was found. There were no within-group or group differences in serum levels of testosterone, cortisol, the ratio of testosterone and cortisol (T/C ratio), GH, leptin, or FGF-21 (Table 2).

### 3.7. Correlations among the Independent Variables

For all subjects, no significant correlations were found among the variables of the changes in aerobic capacity, body composition, and the changes in blood parameters.

## 4. Discussion

This study showed that five weeks of HIIT, despite involving half of the time and exercise energy expenditure when compared to MICT, resulted in a similar improvement in aerobic capacity but had no influence on fat mass or lean mass in the trunk or abdomen. HIIT subjects seemly lost less lean body mass and lean leg mass than did MICT subjects after training. Moreover, both short-term training protocols resulted in a trend to decrease fasting serum glucose but had no effects on systemic hormones, including leptin, testosterone, cortisol, GH, and FGF-21, in overweight and obese young women.

Different forms of HIIT have been shown to significantly increase $\dot{V}O_{2peak}$ [5, 6, 8, 10, 11, 13, 33] and aerobic capacity [10, 11]. The present study found that, after five weeks of this low-volume HIIT protocol, the relative $\dot{V}O_{2peak}$ and PPO were increased by 7.9% and 13.8%, respectively, consistent with an average of 7.3 ± 4.8% increment in $\dot{V}O_{2peak}$ reported in a meta-analysis after the Wingate-based sprint interval intervention in sedentary female cohorts [29]. In accordance with previous studies [5, 17], our study also did not find any additional effect caused by the HIIT protocol when compared to MICT. Moreover, previous studies have demonstrated that, using the same HIIT protocol, $\dot{V}O_{2peak}$ was improved by 15.0% (+5.2 mL·min^−1^·kg^−1^) in obese men for 12 weeks [10] and was increased by 23.8% (+7.6 mL·min^−1^·kg^−1^) in sedentary women for 15 weeks [5], respectively. Although the 7.9% (+2.5 mL·min^−1^·kg^−1^) magnitude of $\dot{V}O_{2peak}$ was relatively smaller, which might be caused by the shorter duration and differences in exercise intensity, this present study indicates that short-term training with this brief HIIT protocol could also result in rapid adaptation in cardiovascular function in inactive obese young women. The possible reasons might be attributed to the upregulated mitochondrial oxidative enzyme activity [7, 8, 21, 34], the enhanced fractional muscle oxygen extraction [6, 35], and/or the increased stroke volume [36].

In addition, HIIT is perceived to be easier than MICT. Since the HR monitored during exercises were analogous (164 ± 8 bpm in HIIT versus 160 ± 12 bpm in MICT) between the two groups, the significantly lower RPE reported in the HIIT group may be mainly caused by the interval exercise mode with submaximal exercise intensity, which was 89% of $\dot{V}O_{2peak}$ during the exercise phases and 76% of $\dot{V}O_{2peak}$ during the recovery phases according to the data measured in the first HIIT session. The game-like nature of HIIT, varying between short sprints and recovery intervals, may be helpful in reducing the perception of effort. Collectively, compared to MICT, the present HIIT protocol is a more time-efficient and easier exercise mode for improving cardiorespiratory fitness in the overweight female cohort.

To our surprise, the MICT group lost 1.7 kg of total lean mass and 0.6 kg of leg lean mass after training. DXA, as a frequently used method to assess body composition, had the smallest detectable differences, at 1.39 kg and 1.30 kg for fat and lean mass in obese children [37], and the CVs for fat and lean mass were 1.2% and 1.1% in obese females [38]. Based on this evidence, we estimate that an approximate 1 kg reduction in total and leg lean mass in the MICT group is probably a consequence of measurement error from the DXA. Moreover, we did not detect any significant reductions in total and regional fat mass as well as fasting leptin levels following the five-week HIIT training intervention. Trapp et al. [5] showed that 15 weeks of HIIT training with a similar protocol significantly reduced resting leptin levels, and the decreases in leptin levels were positively correlated with the decreases in body weight among normal-weight females. On the contrary, a recent study demonstrated that there were no changes in fasting serum leptin despite improvement in body composition after ten weeks of high-intensity interval training in young women with polycystic ovary syndrome [39]. However, due to the body composition, as assessed using a bioelectrical impedance analysis in their study, we believed the notion that short-term exercise training (≤12 weeks) does not affect leptin levels [40] and that long-term exercise training that has reduced leptin levels is generally not independent of changes in body fat mass [41]. Furthermore, given that fat losses were reported with the same 8 s/12 s protocol but using longer interventions (i.e., 15 weeks and 12 weeks) [5, 10], we speculated that, for a less intense HIIT protocol, a longer duration is essential for accumulating measurable alterations in fat loss.

There is no definite conclusion regarding whether HIIT intervention improves body composition in overweight and obese individuals. Several recent studies have shown that HIIT interval training reduces total fat mass [5, 10, 12] and abdominal and visceral fat mass [10, 12] and improves lean mass [5, 8, 10, 12] effectively in both obese and nonobese adults, whereas some evidence reported no changes in body composition in overweight individuals [16, 17] or in active men [42]. However, SIT, a form of supermaximal exercise intensity of a shorter duration [3, 4], seems to be more effective compared to HIIT for improving body composition. A short Wingate-based SIT, which lasted for two weeks, has been shown to reduce abdominal and subcutaneous fat mass in sedentary overweight/obese men, reflected by decreases in waist (−1.4 cm or −1.1%) and hip (−1.1 cm or −1.0%) circumferences [13]. Consistently, six weeks of running SIT interventions led to significant decrement of fat mass and increment of lean mass in recreationally active men [6] and women [11], and the improvements in body composition were comparable to that of MICT [6]. The similar fat losses between HIIT and MICT may result from the increased excess postexercise oxygen consumption (EPOC) [43, 44] and/or the improved muscle oxidative capacity [7, 8, 21], though HIIT had a lower total training volume.

Additionally, in the present study, the training intervention demonstrated a trend toward improved fasting glucose concentrations in the obese female cohort with normal fasting glucose level. Previous studies showed that short-term HIIT, and even acute HIIT, can rapidly improve glucose control in prediabetic [45, 46] and type 2 diabetic patients [21]. On the contrary, some studies reported that, when compared to baseline, short-term Wingate-based HIIT improved insulin sensitivity but had no substantial advantage for improving fasting blood glucose in healthy sedentary [47] and overweight and obese men [13]. Nybo et al. [42] found that 12 weeks of 20-minute high-intensity interval running per week had a similar effect of improving fasting glucose as 60-minute continuous running at 65%  $\dot{V}O_{2peak}$ in sedentary overweight and obese males. Taken together, the discrepancy in fasting blood glucose resulting from HIIT may be attributable to differences in protocols, intervention durations, and initial fasting glucose levels.

We did not find any changes in the basal levels of FGF-21 after the HIIT or MICT interventions in this population. Accumulated evidence derived primarily from animal models indicates that this novel myokine has therapeutic potential for the treatment of type 2 diabetes [48] and has beneficial effects on metabolic disorders [49]. Animal studies have demonstrated that both acute exercise [48] and chronic exercise training [50] could increase serum FGF-21 levels in rodents, and the increment is accompanied by increasing serum levels of ketone bodies, glycerol, and free fatty acids [48]. Among the few studies examining the effects of exercise/training on FGF-21 levels in humans, it has been shown that a single bout of treadmill running exercise [48] as well as two weeks of daily supervised training [23] increased serum FGF-21 levels in healthy men and women.

In the present study, neither exercise regimen had an effect on fasting levels of serum testosterone, cortisol, T/C ratio, and GH, indicating that short-term exercise training, even at a high intensity, cannot induce significant effects on the resting hormones in inactive overweight and obese young women. Although an acute bout of high-intensity interval exercise or sprint exercise would result in marked increment in cortisol and GH levels [27], previous studies have shown that the resting levels of cortisol, testosterone, and GH are unlikely to be influenced by exercise training [24, 51]. Similar to our study, the fasting levels of GH were unaffected by four to six weeks of HIIT/SIT in sedentary men [52] or recreationally active males [53]. Given that the hormonal changes respond mainly to acute exercise [24, 51], future studies should examine the acute responses of different hormones, as well as body composition, before and after HIIT intervention in overweight and obese individuals.

This study has several limitations. First, on the basis of sample estimation from the potential changes of $\dot{V}O_{2peak}$, we acknowledged that the small sample size in the present study limits the ability to draw a meaningful conclusion regarding the efficacy of HIIT in improving body composition against traditional continuous exercise. Second, this study was conducted during a time of the year that people are more likely to gain weight, which started from mid-October and ended in early December. The seasonal factors may also have an influence on body composition since an average net weight gain of up to 0.5 kg in the fall and winter has been reported in a previous study [54]. Because of this, for future studies aimed at reducing weight, seasonal factors should be taken into consideration, and a nonexercise control group is needed for interpretation of the relative results. Finally, considering the great effect of the combination of a hypoenergetic diet and exercise on weight loss and preserving muscle mass [55], future studies may consider managing the factors involving demographic characteristics (gender, age, menstrual cycle, etc.), HIIT modality (low and high demanding), daily physical activity (intensity and amount), and nutrition status (high- and low-protein diet) thoroughly. This would be helpful to ascertain the impact of high-intensity interval training on metabolic outcomes and the potential roles of hormone meditation during changes in overweight and obese populations.

In conclusion, the present study shows that when compared to MICT, short-term brief HIIT intervention with 8 s of high-intensity interval cycling interspersed with 12 s of rest is a more time-efficient approach and is perceived as being easier for improving aerobic fitness and blood glucose in sedentary overweight and obese young women. Neither short-term HIIT nor the MICT intervention had an effect on body composition or the relevant systemic hormones.

## Figures and Tables

**Table 1:** Outcome measures before and after five weeks of exercise training.

	HIIT (*n* = 10)			MICT (*n* = 8)			Time effect		Interaction effect	
	Pre	Post	Δ%	Pre	Post	Δ%	*p*	*η* ^2^	*p*	*η* ^2^
Age (y)	19.8 ± 0.8			19.9 ± 2.1						
Height (m)	163.5 ± 4.7			162.3 ± 5.4						
Weight (kg)	68.3 ± 8.9	69.0 ± 8.9	1.1 ± 2.3	69.1 ± 8.8	68.2 ± 7.8	−1.1 ± 2.5	0.872	0.002	0.087	0.172
BMI (kg·m^−2^)	25.5 ± 2.1	25.7 ± 2.2	1.1 ± 2.3	26.2 ± 2.4	25.9 ± 2.2	−1.1 ± 2.5	0.921	0.001	0.085	0.174
TLM (kg)	41.4 ± 4.6	41.4 ± 4.7	−0.2 ± 2.0	42.9 ± 4.9	41.3 ± 4.9	−3.9 ± 3.3	0.005	0.401	0.008	0.365
Trunk LM (kg)	18.1 ± 2.4	18.2 ± 2.4	0.7 ± 2.2	18.1 ± 3.3	18.1 ± 2.2	2.2 ± 18.8	0.870	0.002	0.857	0.002
Abdomen LM (kg)	8.0 ± 1.3	8.0 ± 1.0	1.1 ± 6.3	7.9 ± 1.3	7.7 ± 1.0	−2.3 ± 6.5	0.499	0.029	0.337	0.058
Leg LM (kg)	16.4 ± 1.9	16.4 ± 1.7	−0.1 ± 3.5	17.0 ± 2.1	16.4 ± 2.0	−3.3 ± 3.0	0.031	0.260	0.063	0.199
TFM (kg)	25.3 ± 6.0	26.0 ± 6.4	2.8 ± 6.5	25.1 ± 4.9	25.3 ± 4.0	1.8 ± 7.0	0.221	0.092	0.514	0.027
Trunk FM (kg)	12.8 ± 3.0	13.1 ± 3.4	1.7 ± 8.9	12.7 ± 2.9	12.7 ± 2.6	0.5 ± 6.6	0.597	0.018	0.517	0.027
Abdomen FM (kg)	4.6 ± 1.2	4.6 ± 1.4	1.0 ± 11.0	4.4 ± 1.0	4.3 ± 0.9	−1.0 ± 8.2	0.965	0.000	0.469	0.033
Leg FM (kg)	8.2 ± 2.9	8.3 ± 2.9	2.3 ± 5.7	8.2 ± 2.1	8.2 ± 1.7	0.4 ± 6.9	0.679	0.011	0.379	0.045
TBF (%)	36.7 ± 4.8	37.4 ± 5.6	1.7 ± 5.0	36.1 ± 3.8	37.0 ± 3.2	2.9 ± 6.0	0.098	0.160	0.773	0.005
Trunk BF (%)	18.6 ± 2.2	18.7 ± 3.0	0.5 ± 7.5	18.3 ± 2.4	18.5 ± 2.1	1.5 ± 5.0	0.465	0.028	0.013	0.001
Abdomen BF (%)	6.6 ± 0.9	6.6 ± 1.3	0.0 ± 10.9	6.2 ± 0.7	6.2 ± 0.8	0.1 ± 7.7	0.949	0.000	0.961	0.000
Leg BF (%)	11.9 ± 3.0	12.0 ± 3.0	1.2 ± 4.4	11.9 ± 2.2	12.0 ± 2.0	1.4 ± 6.0	0.397	0.045	0.975	0.000
$\dot{V}$O_2peak_: (mL·min^−1^·kg^−1^)	34.1 ± 5.7	36.6 ± 6.6	7.9 ± 13.5	34.2 ± 4.3	38.2 ± 6.5	11.7 ± 13.7	0.006	0.380	0.502	0.029
PPO (w)	134.2 ± 23.7	150.2 ± 24.2	13.8 ± 14.8	127.9 ± 17.5	155.5 ± 25.0	21.9 ± 15.4	<0.001	0.61	0.204	0.099

Observed values are expressed as means ± standard deviation. HIIT: high-intensity interval training, MICT: moderate-intensity continuous training, delta (Δ) for the change from before to after intervention, BMI: body mass index, TLM: total lean mass, TFM: total fat mass, TBF (%): percentage of total body fat, and $\dot{V}$O_2peak_: peak oxygen uptake. Partial *η* ^2^ value for effect size (ES).

**Table 2:** Effect of training intervention on blood parameters.

	HIIT (*n* = 10)			MICT (*n* = 8)			Time effect		Interaction effect	
	Pre	Post	Δ%	Pre	Post	Δ%	*p*	*η* ^2^	*p*	*η* ^2^
Glucose (mmol·L^−1^)	4.5 ± 0.2	4.4 ± 0.4	–2.5 ± 8.4	4.6 ± 0.5	4.4 ± 0.6	–4.4 ± 4.9	0.062	0.213	0.640	0.015
Leptin (pg·mL^−1^)	479 ± 29	503 ± 34	106.6 ± 249.8	476 ± 19	491 ± 37	–31.5 ± 39.6	0.131	0.145	0.700	0.010
Testosterone	14.5 ± 6.0	10.9 ± 3.0	112.0 ± 278.8	11.8 ± 4.8	10.0 ± 3.3	–33.7 ± 39.3	0.118	0.155	0.614	0.017
Cortisol (ng·dL^−1^)	428 ± 117	395 ± 118	5.6 ± 11.2	374 ± 138	307 ± 120	3.3 ± 10.1	0.240	0.091	0.692	0.011
T/C ratio (×10^−3^)	31 ± 9.7	26.9 ± 10.1	–9.5 ± 51.9	40.7 ± 36.5	35.5 ± 11.2	–64 ± 39.6	0.567	0.024	0.942	0.000
GH (pg·mL^−1^)	3.1 ± 4.0	2.3 ± 2.6	–6.4 ± 20.4	2.7 ± 1.9	1.6 ± 1.5	0.5 ± 62.3	0.203	0.106	0.872	0.002
FGF-21 (ng·mL^−1^)	0.5 ± 0.4	0.5 ± 0.4	3.0 ± 81.1	0.6 ± 0.7	0.6 ± 0.6	50.4 ± 134.9	0.898	0.001	0.739	0.008

Observed values are expressed as means ± standard deviation. HIIT: high-intensity interval training, MICT: moderate-intensity continuous training, delta (Δ) for the change from before to after intervention, T/C ratio: testosterone to cortisol ratio, GH: growth hormone, and FGF-21: fibroblast growth factor 21. Partial *η* ^2^ value for effect size (ES).

**Table 3:** Carbohydrate, fat, and protein levels before, during, and after training.

Group	CHO (%)	FAT (%)	PRO (%)	Energy intake (kcal·day^−1^)
HIIT before training	51.3 ± 11.7	33.2 ± 8.9	16.2 ± 5.2	2111 ± 690.3
MICT before training	55.2 ± 8.5	28.7 ± 8.1	16.3 ± 4.5	2151 ± 808.3
HIIT training	49.5 ± 11.9	37.0 ± 9.8	14.0 ± 3.9	1867 ± 704.6
MICT training	55.5 ± 9.7	29.8 ± 9.5	15.4 ± 2.3	2309 ± 456.4
HIIT after training	48.1 ± 8.4	36.2 ± 5.4	15.6 ± 3.3	2181 ± 1072.1
MICT after training	51.7 ± 8.1	32.3 ± 5.8	15.9 ± 6.7	2163 ± 786.8

Observed values are expressed as means ± standard deviation. HIIT: high-intensity interval training, MICT: moderate-intensity continuous training, CHO: carbohydrate, and PRO: protein.
